# Longitudinal Analysis of Pulmonary Function Impairment One Year Post-COVID-19: A Single-Center Study

**DOI:** 10.3390/jpm13081190

**Published:** 2023-07-26

**Authors:** Noemi Suppini, Ovidiu Fira-Mladinescu, Daniel Traila, Alexandru Catalin Motofelea, Monica Steluta Marc, Diana Manolescu, Emanuela Vastag, Ram Kiran Maganti, Cristian Oancea

**Affiliations:** 1 Discipline of Pulmonology, “Victor Babes” University of Medicine and Pharmacy Timisoara, 300041 Timisoara, Romania; noemi.suppini@umft.ro (N.S.); traila.daniel@umft.ro (D.T.); marc.monica@umft.ro (M.S.M.); emanuela.tudorache@umft.ro (E.V.); oancea@umft.ro (C.O.); 2 Center for Research and Innovation in Precision Medicine of Respiratory Diseases (CRIPMRD), “Victor Babes” University of Medicine and Pharmacy Timisoara, 300041 Timisoara, Romania; alexandru.motofelea@umft.ro (A.C.M.); dmanolescu@umft.ro (D.M.); 3 Doctoral School, “Victor Babes” University of Medicine and Pharmacy Timisoara, 300041 Timisoara, Romania; 4 Department of Radiology and Medical Imaging, “Victor Babes” University of Medicine and Pharmacy Timisoara, 300041 Timisoara, Romania; 5 School of General Medicine, Sri Devaraj Urs Academy of Higher Education and Research, Karnataka 563103, India; ramkiran.maganti11@gmail.com

**Keywords:** post-acute COVID-19 syndrome, long COVID, pulmonary function tests, lung diseases

## Abstract

Persistent pulmonary impairment post-COVID-19 has been reported, albeit variably. This single-center observational study aims to longitudinally evaluate pulmonary function in 140 COVID-19 survivors one year after recovery, assessing associations with disease severity and pre-existing lung conditions. Participants aged 18 and older, with confirmed SARS-CoV-2 infection, were evaluated using spirometry and Diffusion Capacity of Lungs for Carbon Monoxide (DLCO) tests. Pulmonary function parameters like Forced Expiratory Volume at 1 s (FEV1), Forced Vital Capacity (FVC), and Total Lung Capacity (TLC) were measured. Participants were stratified by age, gender, body mass index, smoking status, and lung damage severity via computed tomography (CT). The cohort consisted of mostly males (58.6%), with a mean age of 53.8 years and body mass index of 24.9 kg/m^2^. Post-COVID fibrosis was seen in 22.7%, 27.3%, and 51.9% of mild, moderate, and severe disease patients, respectively (*p* = 0.003). FVC significantly reduced with disease severity (*p* < 0.001), while FEV1, FEF25-75, and DLCO showed a non-significant downward trend. FEV1/FVC ratio increased with disease severity (*p* = 0.033), and TLC and RV significantly declined (*p* = 0.023 and *p* = 0.003, respectively). A one-year follow-up indicated a non-significant change in FVC, FEV1, FEV1/FVC ratio, FEF25-75, and RV compared with the 40-day measurement, but it revealed significant improvements in DLCO and TLC (*p* = 0.010). There were significant mean increases in FVC, FEV1, DLCO, TLC, and RV across all disease severities over one year. They were most pronounced in the patients with a history of severe COVID-19, who had a better recovery over one year, compared with the mild and moderate COVID-19 patients whose lung function almost normalized. One year after the SARS-CoV-2 infection, we observed a significant association between disease severity and post-COVID fibrotic changes. Though some lung function parameters remained stable over the year, significant improvements were noted in DLCO and TLC. Particularly, individuals with severe disease showed substantial recovery in lung function, indicating the potential reversibility of COVID-19-related pulmonary damage.

## 1. Introduction

Coronavirus disease 2019 (COVID-19) has spread throughout the world since its initial discovery in Wuhan, China, in December 2019 [1]. More than 6.8 million people have died as a result of COVID-19 diagnoses in more than 700 million people all over the world in recent years [2]. Due to the massive worldwide disease burden, COVID-19 continues to be a public health issue of concern on a global scale. In addition to the acute illness, long-term effects of COVID-19 have also been identified, and patients may experience recurrent symptoms, deteriorated lung function, and consequences that are both pulmonary and systemic [3].

Evidence indicates that COVID-19 is most likely to affect the lungs, inducing a variety of pathophysiological modifications such as diffuse damage to the alveolar epithelium, formation of hyaline membrane, and in addition, damage of capillaries that will result in bleeding. Furthermore, alveolar septal fibrous proliferation and pulmonary consolidation were observed [4]. The severe damage to alveolar cells, along with subsequent fibro-proliferation, is a hallmark of COVID-19 and suggests a possibility for persistent vascular and alveolar remodeling that could result in lung fibrosis and/or pulmonary hypertension [5]. These findings raise questions about how patients who have been discharged should be assessed for lung injury. The most often used objective functional respiratory assessments are pulmonary function tests (PFTs) [6].

Furthermore, the abnormalities found on chest computed tomography (CT), as stated in epidemiological data, might cause pulmonary fibrosis, and they can be examined alongside pulmonary function [7]. Pulmonary function testing 12 weeks after discharge is advised for patients with severe forms of COVID-19 pneumonia, according to clinical guidelines. Furthermore, pulmonary function evaluation must be performed following abnormal chest X-rays in cases of pneumonia [8]. According to the earliest findings on COVID-19-related lung function, patients can present a restrictive defect and small airway dysfunction that can be permanent and are unrelated to the severity of the disease [9].

Moreover, studies found restrictive patterns followed by a reduction in diffusion capacity, both connected to the severity of the illness [4]. The prevalence of fibrotic lesions will emerge over time, but the discharge assessments of patients with COVID-19 suggest an increased proportion of fibrotic-type lung function abnormalities [4]. These reports are consistent with the literature on previous coronavirus infections, such as severe acute respiratory syndrome (SARS) and Middle East respiratory syndrome (MERS), which also indicates that patients may continue to exhibit persistent impairment for months or even years after being discharged [10,11,12]. The variability of the existing data emphasizes the necessity for larger cohort studies to elucidate the features of COVID-19 consequences. Therefore, the aim of this study is to characterize the pulmonary function in a cohort of COVID-19 survivors one year after recovery and analyze the association between the severity of the acute COVID-19 course and previous lung disease.

## 2. Materials and Methods

### 2.1. Design and Ethics

We conducted an observational study to evaluate and monitor the pulmonary function of 140 post-COVID patients who received care at our clinic. Specifically, we assessed spirometry and Diffusing Capacity of Lungs for Carbon Monoxide (DLCO) to obtain the following parameters: Forced Expiratory Volume at 1 s (FEV1), Forced Vital Capacity (FVC), FEV1/FVC ratio, Forced Mid-expiratory Flow (FEF 25-7), DLCO, Residual Volume (RV), and Total Lung Capacity (TLC). Patients were stratified by age, gender, body mass index (according to World Health Organization criteria [13]), smoking status, and lung damage severity evaluated with computed tomography (CT).

The study was conducted in accordance with the Declaration of Helsinki and approved by the Institutional Review Board of the “Victor Babes” Hospital for Infectious Diseases and Pulmonology, from Timisoara, Romania (approval number 1136).

### 2.2. Inclusion and Exclusion Criteria

The inclusion criteria for this study were as follows. Participants must be aged 18 years or older at the time of recruitment. Only individuals who have had a confirmed infection with the SARS-CoV-2 virus were considered for inclusion. This confirmation of infection must have been established with a positive RT-PCR or antigen test. The diagnosis of COVID-19 infection must have occurred within 40 days prior to the recruitment period (the initial measurement). It was required that participants were clinically stable at the time of recruitment, presenting no acute symptoms of the infection. All participants needed to be physically capable of performing the required pulmonary function tests, including spirometry and DLCO, without severe discomfort or risk. All patients were required to attend both appointments 40 days after SARS-CoV-2 infection clearance and after 1 year. Finally, all participants were required to provide informed consent prior to participation in the study.

The exclusion criteria for the study were specifically set to ensure accurate results and participant safety. Participants who have not experienced an infection with the SARS-CoV-2 virus or have not been tested for the virus were not considered for this study. Additionally, we excluded individuals who have not undergone the necessary spirometry and DLCO pulmonary function tests at 40 days and after 1 year. Any participants with existing neurological or psychiatric conditions that might interfere with their ability to perform or interpret the pulmonary function tests were also excluded from the study. Similarly, those with unstable medical conditions or any conditions that could place them at increased risk during testing or interfere with the interpretation of results were not included. Patients with severe pre-existing pulmonary diseases, such as advanced chronic obstructive pulmonary disease (COPD), severe asthma, or advanced interstitial lung disease (ILD), were likewise excluded to prevent potential confounding of the pulmonary function results. Lastly, the study required all participants to be capable of understanding and providing informed consent; those unable to do so were excluded.

### 2.3. Definitions and Methods

Comprehensive clinical data were meticulously extracted from the electronic medical records of each participant. This included demographic information (e.g., age, sex, race/ethnicity), lifestyle factors (particularly smoking status), medical comorbidities, and relevant radiographic imaging data such as chest computed tomography (CT) scans and X-rays. We used the Cosmed Quark PFT equipment from the “Victor Babes” Hospital for Infectious Diseases and Pulmonology to perform standardized spirometry and single-breath carbon monoxide uptake tests in the lungs. The quality of these tests was stringently evaluated based on the American Thoracic Society and the European Respiratory Society (ATS–ERS) guidelines [14]. The resulting values were then interpreted according to the ATS–ERS criteria. Parameters were compared to the lower (LLN) and higher (HLN) limit of normal for each individual.

The classification of the severity of patients’ COVID-19 infections was conducted according to World Health Organization (WHO) guidelines [15]. These encompass a spectrum of disease severity: Mild disease included patients with a confirmed COVID-19 diagnosis but without clinical or radiographic evidence of pneumonia or hypoxia. Moderate disease was characterized by the presence of clinical signs of pneumonia but an oxygen saturation above 90% in room air. Severe disease involved patients demonstrating clinical or radiographic evidence of pneumonia accompanied by an oxygen saturation below 90% in room air. Lastly, patients were classified as having critical disease if they developed complications such as acute respiratory distress syndrome (ARDS), sepsis, or septic shock.

### 2.4. Statistical Analysis

For analysis and reporting, the collected PFT data were normalized to population averages and will be presented as percentages of predicted values rather than raw data to facilitate clinical interpretation. Continuous data are presented as a mean with Standard Deviation (SD) where data are normally distributed and as a median with the 25th and 75th centiles for non-parametric data. Categorical data are summarized as frequencies and percentages. The differences between groups for continuous normally distributed data were tested using Welch’s t-test for two groups and using the linear model ANOVA for three or more groups. Non-parametric continuous data were tested using a Mann–Whitney U-test for two groups or using the Kruskal–Wallis test for three or more groups. The differences across categorical data were tested using the χ² test or Fisher’s exact test when expected cell counts were less than five. All statistical analyses were conducted with R (version 3.6.3).

## 3. Results

### 3.1. Background of the Patients

In our study, we longitudinally analyzed the impairment of pulmonary function in 140 patients one year post-COVID-19 infection. The baseline characteristics of these patients are summarized in Table 1. The mean age of the patients was 53.8 years (±13.9 SD), and a majority of them were male (58.6%, n = 82). The average body mass index (BMI) was found to be 24.9 kg/m^2^ (±7.5 SD), indicating a generally healthy weight range.

A significant proportion of the patient cohort was either current or former smokers (56.5%, n = 79), which could have potentially affected their lung function independently of their COVID-19 infection. As presented in Table 1, 36.4% (n = 51) of the patients had a Charlson Comorbidity Index (CCI) of 2 or more, excluding lung disease, which suggests the presence of other underlying health conditions that may complicate their recovery and lung function post-COVID-19. In terms of the severity of their initial COVID-19 illness, the majority of patients had experienced mild disease (47.1%, n = 66). Meanwhile, 15.8% (n = 22) of patients had moderate severity, and 37.1% (n = 52) had severe COVID-19. This diverse distribution allowed us to analyze pulmonary function impairment across a broad spectrum of disease severities.

Table 2 presents the results of lung function measurements conducted 40 days after viral clearance in the three groups of patients with differing initial COVID-19 severity: mild, moderate, and severe. Post-COVID fibrosis was seen in 22.7% (n = 15) of the patients with mild COVID-19, 27.3% (n = 6) of those with moderate disease, and a significant 51.9% (n = 27) of patients with severe disease, which was statistically significant with a *p*-value of 0.003, indicating a strong association between disease severity and fibrotic changes post-recovery. Forced Vital Capacity (FVC) differed significantly among the groups (*p* < 0.001), with mean values decreasing from 97.8 ± 9.6 in the mild group to 89.9 ± 15.0 in the moderate group, and further to 84.9 ± 17.7 in the severe group, suggesting that disease severity was associated with reduced FVC.

Although the Forced Expiratory Volume at 1 s (FEV1) also decreased from the mild to severe groups, this decrease was not statistically significant (*p* = 0.074). Similarly, the Forced Expiratory Flow 25–75% (FEF25-75) and the diffusing capacity of the lungs for carbon monoxide (DLCO) showed a trend toward lower values with increasing disease severity, but these changes were not statistically significant (*p* = 0.059 and *p* = 0.052, respectively). Interestingly, the ratio of FEV1 to FVC increased with disease severity (*p* = 0.033), which might reflect a relative preservation of FEV1 compared to the decline in FVC. Total Lung Capacity (TLC) and Residual Volume (RV) demonstrated significant declines with increasing disease severity (*p* = 0.023 and *p* = 0.003, respectively), indicating that severe COVID-19 might lead to a long-term reduction in these lung function parameters.

### 3.2. Functional Evaluation after One Year

Table 3 reveals the results of the longitudinal analysis of pulmonary function one year post-COVID-19, evaluating the changes in various lung parameters. The Forced Vital Capacity (FVC) showed a minor decline from the initial average of 88.5 ± 18.9 to the final average of 88.1 ± 19.9, but this change was not statistically significant (*p* = 0.840), suggesting that there was not a significant decrease in this parameter over the course of the year. Forced Expiratory Volume in 1 s (FEV1) increased slightly from the initial mean of 88.2 ± 17.2 to the final mean of 89.2 ± 17.3, but again, this change was not statistically significant (*p* = 0.619), indicating that patients’ abilities to exhale forcibly in one second did not significantly change one year after the disease.

**Figure 1:**
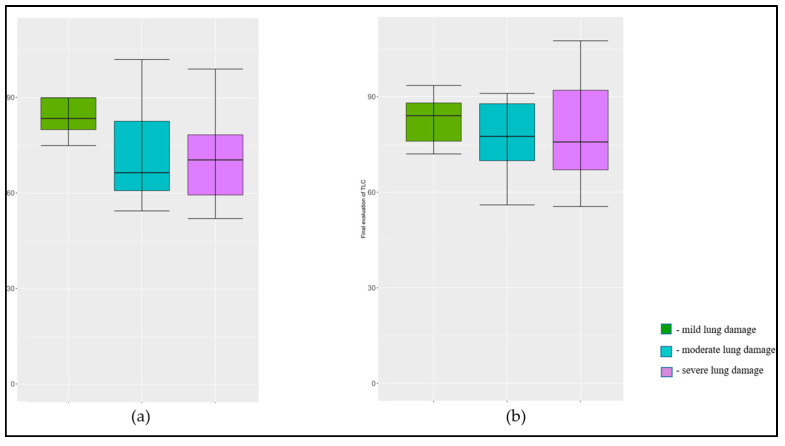
Evolution of TLC in one year after acute COVID-19 disease by lung damage severity; (**a**) initial evaluation (at 40 days after COVID-19); (**b**) final evaluation at one year after COVID-19.

**Figure 2:**
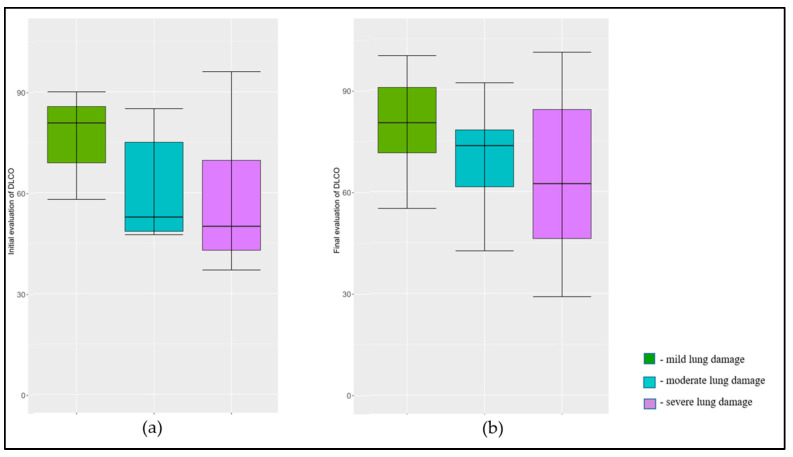
Evolution of DLCO in one year after acute COVID-19 disease by lung damage severity; (**a**) initial evaluation (at 40 days after COVID-19); (**b**) final evaluation at one year after COVID-19.

Similarly, the FEV1/FVC ratio, Forced Expiratory Flow 25–75% (FEF25-75), and Residual Volume (RV) showed no statistically significant changes from the initial to final evaluations with *p*-values of 0.194, 0.862, and 0.185, respectively, suggesting stability in these lung function parameters over time. However, there were significant improvements in the Diffusing Capacity of the Lungs for Carbon Monoxide (DLCO) and Total Lung Capacity (TLC) over the year, as presented in Figure 1 and Figure 2. The mean DLCO improved from 61.7 ± 21.5 initially to 68.9 ± 19.9 finally (*p* = 0.010), and the mean TLC improved from 77.9 ± 21.0 to 83.8 ± 19.0 (*p* = 0.010), indicating statistically significant enhancements in the patients’ lung diffusion capacities for carbon monoxide and total lung capacities one year post-COVID-19.

The lung function of patients one year after the viral clearance of COVID-19 showed significant variations based on the severity of the disease as shown in Table 4. Post-COVID fibrosis was noted in 7.6% of mild cases, 13.6% of moderate cases, and 19.2% of severe cases, although the *p*-value of 0.170 suggests these differences were not statistically significant. The Forced Vital Capacity (FVC) showed significant differences among groups, with a mean of 98.2 ± 9.0 in mild cases, 94.0 ± 13.1 in moderate cases, and 91.7 ± 15.8 in severe cases (*p* = 0.020), indicating a lower mean FVC in severe cases. Forced Expiratory Volume in 1 s (FEV1) trended lower in severe cases with 90.8 ± 13.1, compared to 96.6 ± 13.8 in mild cases and 92.4 ± 10.9 in moderate cases, although the *p*-value of 0.054 suggests this did not reach statistical significance.

The ratio of FEV1 to FVC was the highest in severe cases with a mean of 82.8 ± 7.2, as compared to 78.9 ± 7.0 in mild cases and 80.2 ± 6.4 in moderate cases, with this difference reaching statistical significance (*p* = 0.012). Forced Expiratory Flow at 25–75% of pulmonary volume (FEF25-75) did not differ significantly among the groups (*p* = 0.474). The mean Diffusing Capacity of the Lungs for Carbon Monoxide (DLCO) was the lowest in severe cases at 71.9 ± 17.5, as compared to 78.4 ± 12.9 in mild cases and 76.8 ± 18.3 in moderate cases, but the difference was not statistically significant (*p* = 0.079).

Total Lung Capacity (TLC) was significantly lower in severe cases with a mean of 81.3 ± 19.2, as compared to 89.5 ± 15.3 in mild cases and 85.0 ± 14.4 in moderate cases (*p* = 0.032). Residual Volume (RV), expressed as a percentage of the predicted value, was also lower in severe cases at 77.0 ± 21.6, compared to 86.4 ± 20.2 in mild cases and 83.1 ± 19.7 in moderate cases, but the difference did not reach statistical significance (*p* = 0.051).

Table 5 presents the differences in lung function measurements taken at 40 days and 1 year post-viral clearance in patients with varying severities of COVID-19. The values represented the mean changes in each measurement over the time period, stratified by the severity of the illness. Overall, there was a significant increase in FVC, FEV1, DLCO, TLC, and RV in patients of all severity categories, as suggested by the very small *p*-values (<0.001). The mean increase in FVC was more pronounced in severe cases (6.8 ± 2.9) compared to moderate (4.1 ± 3.0) and mild cases (0.4 ± 1.2). Similar patterns were observed in FEV1, DLCO, TLC, and RV, with the most significant changes occurring in patients who had severe COVID-19. This indicates that over the period of one year, lung functions improved significantly, particularly in patients with severe disease.

The FEV1/FVC ratio, however, decreased in mild and moderate cases (−0.7 ± 2.5 and −1.3 ± 1.9, respectively) while it increased marginally in severe cases (−0.2 ± 1.0). This change was not statistically significant across the groups as indicated by the *p*-value of 0.082. Lastly, FEF25-75 showed an increase across all categories, but this was not statistically significant with a *p*-value of 0.213. This suggests that while there were improvements in this measurement, they were not consistently significant across the different severity groups.

## 4. Discussion

### 4.1. Literature Findings

The present course of recovery for patients infected with the SARS-CoV-2 virus is still under research. Our study showed a correlation between the severity of restrictive patterns and lung function loss in post-COVID patients at a second re-evaluation after one year. Also, patients with severe COVID-19 had a higher incidence of post-viral fibrosis and more significant impairments in most lung function parameters compared to those with mild or moderate disease, only 40 days after viral clearance, confirming a correlation between initial COVID-19 severity and subsequent lung function impairment. However, FEV1, FEF25-75, and DLCO impairments were not statistically significant among the groups (at 40 days), suggesting these measurements might not be sensitive markers for the measurement of lung function impairment post-COVID-19. Another study of computed tomography data in individuals recovering from COVID-19 infection discovered that those who develop fibrotic alterations were more likely to be older and to have a more severe form of the disease [15], which was consistent with our findings.

Regarding the DLCO measurements, our results were consistent with previous studies, which showed that severe forms of COVID-19 were linked to severe functional and radiological abnormalities and a decrease in DLCO at four months of follow-up [16]. Additionally, studies show a significant improvement in lung function at the follow-up, especially in severe forms of the disease [17,18]. According to a study that analyzed pre- and post-infection pulmonary function parameters, published by Kristyn L. Lewis [19], pulmonary function tests in individuals who recover from COVID-19 without intubation or positive pressure ventilation are likely to revert to pre-infection parameters. According to one research, the majority of COVID-19 patients’ chest CT scores for pulmonary fibrosis dramatically decreased one month, two months, and three months after discharge, indicating that post-COVID-19 lung fibrosis is likely to decrease over time [20].

Regarding the long-term effects of SARS-CoV-2 infection, some similarities were consistent with earlier coronavirus outbreaks, specifically the SARS and MERS. However, there is insufficient data to predict the natural course of pulmonary fibrosis in COVID-19. The fibrotic lung alterations in recovered individuals have been observed in long-term trials with SARS and MERS. In a 15-year follow-up study of 71 SARS patients, it was shown that 38% of patients had chronic pulmonary fibrosis following the initial infection, and 4.6% of these patients also had decreased Forced Vital Capacity (FVC), a sign of compromised lung function [12]. In a median of 43 (range 32–320) days after hospital discharge, there is radiographic evidence of lung fibrosis in around one-third of patients, notwithstanding the paucity of follow-up studies on MERS patients [21]. Lung fibrosis may be a potential long-term consequence for COVID-19 patients, given that almost 30% of SARS and MERS survivors have persistent radiological and physiological abnormalities associated with fibrotic lung disease [22]. It will be necessary to do long-term follow-up investigations to determine the real prevalence of post-COVID-19 fibrosis.

Even though patients with chronic pulmonary disease were excluded from our study, according to a systematic review and meta-analysis, individuals with asthma and COVID-19 infection did not have an increased risk of death, hospitalization, or length of hospital stay compared to those without asthma [23]. Surprisingly, although this finding did not achieve statistical significance, there was an observed trend toward a decreased risk of mortality among individuals with asthma [23]. In the case of a severe acute COVID-19 condition, it has been suggested that type 2 (Th2) inflammation may have a protective function. Much research attempted to test this hypothesis [24,25,26].

In the current study, all patients received corticosteroids according to local guidelines, however, dexamethasone has been demonstrated to decrease mortality in individuals with severe COVID-19, and it is probable that inhaled corticosteroids’ systemic absorption may have a beneficial effect on the course of the illness [27]. A number or proportion of patients who were receiving either systemic or inhaled steroids (ICS) at the time of COVID-19 diagnosis was reported by many investigations [28,29,30,31,32]. Another study showed that asthmatic patients treated with ICS and biologic might be associated with a protective effect against severe COVID-19 infection [33].

Although the population of patients in the current study was homogenous and without patients with pre-existing lung disease, it was observed in previous studies that there was an increased likelihood of worse functional outcomes among COVID-19 individuals with underlying ILD due to their lower pulmonary reserve [34]. In these patients, COVID-19 is of special relevance since they are more likely to experience acute exacerbations brought on by a viral infection due to both reduced lung function and a higher risk of such events [34]. Studies suggest that idiopathic pulmonary fibrosis (IPF) patients may be particularly susceptible to COVID-19 because of their high rates of hospitalization, need for ICU care, and death [35,36]. There have been limited investigations of the consequences of COVID-19 infection in IPF patients. Naqvi et al. evaluated the impact of COVID-19 on 251 individuals with IPF in research. The hospitalization rate was 44%, with a 15.9% fatality rate [37]. A study published by Lee H. et al. Revealed that compared to people without ILD, patients with ILD are more prone to COVID-19 and have more severe COVID-19 symptoms [38]. As hypothesized, individuals with ILD had worse outcomes when their condition was more advanced, as shown with pulmonary function tests. [36] Another study published by Drake et al. concluded that the Forced Vital Capacity (FVC) <80% was an independent risk factor for mortality (HR = 1.72, 95%; CI = 1.05–2.83) [39]. Esposito et al. revealed in their study the same effect regarding the Transfer Factor of the Lung for Carbon Monoxide (TLCO) [40]. By implementing the necessary proactive, preventative steps, including vaccination, doctors and patients should be made aware of their specific needs to reduce their risk of exposure to SARS-CoV-2 [41].

### 4.2. Study Limitations

Our investigation, despite its merits, was not without limitations. First, our study might have overlooked potential confounding variables, particularly as we did not assess lung volumes through whole-body plethysmography. The omission of this measurement may lead to an incomplete understanding of the disease’s impact on lung function. Second, the absence of a control group in our study could potentially undermine the strength of our conclusions. A control group of uninfected individuals, matched for age, sex, and other demographic factors, would have provided a benchmark against which to compare the pulmonary function of our post-COVID-19 patients. Third, a significant limitation is the lack of baseline lung function data. The absence of pre-COVID-19 pulmonary function tests limits the extent to which we can infer the direct impact of the disease on patients’ lung functions. This information could have served as a crucial reference point to precisely determine the deterioration or changes caused by COVID-19. Lastly, while our sample size of 140 patients was sufficient for a preliminary analysis, a larger cohort would improve the robustness of our findings. Moreover, a larger sample size could increase the generalizability of findings and reduce bias. A larger sample size would increase the statistical power of the study, reducing the likelihood of Type II errors and allowing for a more nuanced understanding of how COVID-19 impacts patients with different demographic and clinical characteristics.

## 5. Conclusions

Our longitudinal analysis of pulmonary function impairment one year post-COVID-19 revealed significant improvements in several lung function parameters, particularly in patients who had severe disease. While FVC and FEV1 did not change significantly over the year, a declining trend was evident, especially in severe cases. Concurrently, an increase in the FEV1/FVC ratio was significant in severe cases, suggesting a relative preservation of FEV1 compared to the decline in FVC, indicating the role of disease severity at the time of infection in pulmonary function recovery. Although not statistically significant, post-COVID fibrosis was observed in all severity groups, suggesting possible long-term structural changes in the lungs post-infection. Importantly, we observed statistically significant improvements in the DLCO and Total Lung Capacity over the year, pointing to a certain degree of resilience and adaptability of the pulmonary functions. The severity of the initial disease still significantly influenced pulmonary function after one year post-infection, with TLC significantly lower in severe cases, highlighting the enduring impact of severe COVID-19 on lung capacity. Overall, our findings underscore the need for continued monitoring and care for lung health in COVID-19 patients—particularly in those with severe infection—and highlight the complex and enduring impact of COVID-19 on lung function, pointing to the necessity of further longitudinal studies for a comprehensive understanding of long-term COVID-19 outcomes.

## Figures and Tables

**Table 1:** Baseline characteristics of the patients.

Variables	Patients (n = 140)
Age, years (mean ± SD)	53.8 ± 13.9
Sex, male (n,%)	82 (58.6%)
BMI, kg/m^2^ (mean ± SD)	24.9 ± 7.5
Smoking, current/former (n,%)	79 (56.5%)
CCI ≥ 2 (n,%) *	51 (36.4%)
COVID-19 severity	
Mild	66 (47.1%)
Moderate	22 (15.8%)
Severe	52 (37.1%)

*—Excluding lung disease; SD—Standard Deviation; BMI—body mass index; CCI—Charlson Comorbidity Index.

**Table 2:** Lung function measurements 40 days after viral clearance, stratified by COVID-19 severity.

Variables (mean ± SD)	Mild (n = 66)	Moderate (n = 22)	Severe (n = 52)	*p*-Value
Post-COVID fibrosis (n,%)	15 (22.7%)	6 (27.3%)	27 (51.9%)	0.003
FVC% (% pred.)	97.8 ± 9.6	89.9 ± 15.0	84.9 ± 17.7	<0.001
FEV1% (% pred.)	95.4 ± 18.2	90.3 ± 15.8	86.1 ± 17.6	0.074
FEV1/FVC (%)	83.0 ± 9.1	81.5 ± 5.1	79.6 ± 5.7	0.033
FEF 25–75% (% pred.)	81.0 ± 5.1	79.6 ± 6.3	78.5 ± 6.0	0.059
DLCO (% pred.)	77.9 ± 17.2	74.5 ± 20.1	66.7 ± 22.5	0.052
TLC (% pred.)	88.0 ± 18.9	83.2 ± 16.0	76.6 ± 20.8	0.023
RV (% pred.)	84.4 ± 24.2	76.9 ± 20.0	67.0 ± 21.4	0.003

SD—Standard Deviation; FVC (Forced Vital Capacity); FEV1 (Forced Expiratory Volume in 1 s); FEF25-75 (Forced Expiratory Flow 25–75%); DLCO (Diffusing Capacity of the Lungs for Carbon Monoxide); TLC (Total Lung Capacity); and RV (Residual Volume).

**Table 3:** Evaluation of the lung parameters after one year in the entire cohort.

N = 140		FVC %pred.	FEV1 %pred.	FEV1/FVC %	FEF25-75 %pred.	DLCO %pred.	TLC %pred.	RV %pred.
Mean ± SD	40 days *	88.5 ± 18.9	88.2 ± 17.2	82.0 ± 6.4	86.0 ± 24.9	61.7 ± 21.5	77.9 ± 21.0	73.1 ± 26.1
	1 year *	88.1 ± 19.9	89.2 ± 17.3	82.6 ± 6.7	86.4 ± 26.4	68.9 ± 19.9	83.8 ± 19.0	75.8 ± 24.8
*p*-value		0.840	0.619	0.194	0.862	0.010	0.010	0.185

*—Compared using Paired Samples T-Test; SD—Standard Deviation; FVC (Forced Vital Capacity); FEV1 (Forced Expiratory Volume in 1 s); FEF25-75 (Forced Expiratory Flow 25–75%); DLCO (Diffusing Capacity of the Lungs for Carbon Monoxide); TLC (Total Lung Capacity); and RV (Residual Volume).

**Table 4:** Lung function measurements 1 year after viral clearance, stratified by COVID-19 severity.

Variables (Mean ± SD)	Mild (n = 66)	Moderate (n = 22)	Severe (n = 52)	*p*-Value
Post-COVID fibrosis (n,%)	5 (7.6%)	3 (13.6%)	10 (19.2%)	0.170
FVC% (% pred.)	98.2 ± 9.0	94.0 ± 13.1	91.7 ± 15.8	0.020
FEV1% (% pred.)	96.6 ± 13.8	92.4 ± 10.9	90.8 ± 13.1	0.054
FEV1/FVC (%)	78.9 ± 7.0	80.2 ± 6.4	82.8 ± 7.2	0.012
FEF 25–75% (% predicted)	89.3 ± 16.2	84.1 ± 14.0	87.3 ± 20.3	0.474
DLCO (% pred.)	78.4 ± 12.9	76.8 ± 18.3	71.9 ± 17.5	0.079
TLC (% pred.)	89.5 ± 15.3	85.0 ± 14.4	81.3 ± 19.2	0.032
RV (% pred.)	86.4 ± 20.2	83.1 ± 19.7	77.4 ± 21.6	0.051

SD—Standard Deviation; FVC (Forced Vital Capacity); FEV1 (Forced Expiratory Volume in 1 s); FEF25-75 (Forced Expiratory Flow 25–75%); DLCO (Diffusing Capacity of the Lungs for Carbon Monoxide); TLC (Total Lung Capacity); and RV (Residual Volume).

**Table 5:** Mean differences in lung function measurements 40 days and 1 year after viral clearance, stratified by COVID-19 severity.

Variables (Mean ± SD)	Mild (n = 66)	Moderate (n = 22)	Severe (n = 52)	*p*-Value
FVC% (% pred.)	0.4 ± 1.2	4.1 ± 3.0	6.8 ± 2.9	<0.001
FEV1% (% pred.)	1.2 ± 0.9	2.1 ± 1.6	4.7 ± 1.4	<0.001
FEV1/FVC (%)	−0.7 ± 2.5	−1.3 ± 1.9	−0.2 ± 1.0	0.082
FEF 25–75% (% pred.)	8.3 ± 10.6	4.5 ± 8.3	8.8 ± 9.6	0.213
DLCO (% pred.)	0.5 ± 1.4	2.3 ± 1.0	5.2 ± 3.3	<0.001
TLC (% pred.)	1.5 ± 0.6	1.8 ± 0.9	4.7 ± 1.5	<0.001
RV (% pred.)	2.0 ± 3.1	6.2 ± 5.5	10.4 ± 7.0	<0.001

SD—Standard Deviation; FVC (Forced Vital Capacity); FEV1 (Forced Expiratory Volume in 1 s); FEF25-75 (Forced Expiratory Flow 25–75%); DLCO (Diffusing Capacity of the Lungs for Carbon Monoxide); TLC (Total Lung Capacity); RV (Residual Volume).

## Data Availability

Data is available upon request.
